# Interactive Effects of Genetic Susceptibility and Early-Life Tobacco Smoke Exposure on the Asthma–Eczema Complex Phenotype in Children: 6-Year Follow-Up Case-Control Study

**DOI:** 10.3390/ijms27010346

**Published:** 2025-12-29

**Authors:** Anna Dębińska, Hanna Danielewicz, Anna Drabik-Chamerska, Barbara Sozańska

**Affiliations:** Department and Clinic of Paediatrics, Allergology and Cardiology, Wrocław Medical University, ul. Chałubińskiego 2a, 50-368 Wrocław, Poland

**Keywords:** gene–environment interaction, *TNS1*, *NRXN1*, eczema, asthma, ETS exposure, tobacco smoke, polymorphism, single nucleotide, complex phenotype

## Abstract

Atopic eczema and asthma frequently co-occur, forming a distinct complex phenotype that likely arises from shared genetic pathways and early-life environmental influences. We aimed to investigate whether variants in *TNS1* and *NRXN1*—previously identified in a genome-wide interaction study—influence susceptibility to atopic eczema and the asthma–eczema phenotype and whether early-life environmental tobacco smoke (ETS) exposure modifies these genetic effects. A total of 188 Caucasian children under 2 years at recruitment were prospectively followed up to 6 years of age. Eligibility of all participants for the study or control group was based on a questionnaire and a physician-confirmed diagnosis of eczema and asthma. Early-life ETS exposure was assessed by parental questionnaire. All participants were genotyped for *TNS1* and *NRXN1* SNPs. The *TNS1* rs918949 [T] allele was associated with the combined asthma–eczema phenotype but not with eczema alone. Synergistic gene–environment interactions were identified for both *TNS1* and *NRXN1*, with the highest risk of the combined asthma–eczema phenotype observed among ETS-exposed carriers of risk alleles. Our findings provide the first independent replication of evidence suggesting that *TNS1* and *NRXN1* may contribute to the asthma–eczema comorbidity through mechanisms that could be substantially modified by early-life ETS exposure.

## 1. Introduction

Atopic diseases, such as eczema and asthma, represent the most common chronic conditions in children and are closely associated with one another [1,2,3,4]. This co-occurrence suggests shared pathogenic mechanisms and overlapping genetic determinants, as evidenced by their strong familial aggregation and frequent coexistence of both conditions within the same individuals [5,6,7,8]. Atopic eczema often predates the onset of other allergic diseases and is a well-established risk factor for subsequent progression to asthma, particularly in cases with early-onset atopic eczema [9,10,11]. One of the most compelling objectives of current research is to identify risk factors associated with atopic eczema that may also serve as predictors of subsequent atopic disease progression. However, the mechanisms underlying the development of atopic eczema and its subsequent progression to asthma remain incompletely understood. Linkage and association studies have identified multiple shared inherited susceptibility variants that contribute to the increased risk of both eczema and asthma [12,13,14,15]. Nonetheless, these variants account for only a fraction of the observed heritability, as most studies have focused on broad allergic disease phenotypes defined as the presence of any one of the allergic conditions, without addressing the complex phenotype of asthma–eczema comorbidity, which may better reflect the natural history of allergic disease progression.

Given that the co-occurrence of asthma and eczema appears to represent a distinct phenotype with a unique pathogenic pathway, it was of particular interest to investigate genetic variants specifically associated with this comorbidity. The first meta-analysis of genome-wide association studies (GWAS) addressing the comorbidity of asthma and eczema within the context of atopic march reported several genes already implicated in either asthma or eczema, along with two new loci associated with allergic diseases for the first time [16]. More recently, a second GWAS meta-analysis aimed to detect genes uniquely contributing to the combined asthma–eczema phenotype, resulting in the discovery of six new genes not previously linked to allergic diseases [17]. Nevertheless, genetic predisposition alone cannot fully account for the susceptibility and phenotypic heterogeneity of multifactorial diseases such as eczema and asthma, which result from complex interactions between multiple genetic and environmental factors.

Among the environmental factors, early-life exposure to environmental tobacco smoke (ETS) warrants particular attention due to its well-documented effects on airway inflammation, lung function, and immune regulation [18,19,20]. A substantial body of evidence demonstrates that both prenatal and postnatal exposure to ETS represents a significant risk factor for wheezing and asthma [21,22] and, to a lesser extent, for eczema, although studies have yielded conflicting results regarding the latter association [23,24,25]. Importantly, children with a genetic predisposition to atopy may exhibit heightened susceptibility to the detrimental effects of tobacco smoke, suggesting potential gene–environment interactions [26,27,28]. However, the full extent to which ETS influences genetic susceptibility to increase disease risk is not yet completely understood. Although individual associations between genetic variants and atopic diseases [29,30,31,32,33,34], as well as between ETS exposure and allergic outcomes [21,24,35], are well established, research exploring their combined effects, particularly in relation to the asthma–eczema comorbidity, remains limited. To our knowledge, only one genome-wide interaction study (GWIS) investigating the combined asthma–eczema phenotype in the context of ETS exposure has been published to date. This analysis identified two previously unreported candidate genes, *NRXN1* (2p16) and *TNS1* (2q35), showing significant interactions with environmental tobacco smoke (ETS) exposure [36]. These findings highlight the importance of investigating distinct phenotypes, such as comorbid conditions and gene–environment interactions, to identify new genetic susceptibility loci, as well as the need for replication analysis in independent populations [36]. Understanding whether genetic predisposition and early-life ETS exposure interact in the development of the asthma–eczema phenotype may provide insights into the shared pathophysiological mechanisms underlying these conditions and inform strategies for early prevention in genetically predisposed populations.

Therefore, the present study aimed to investigate the role of SNPs in the *NRXN1* and *TNS1* genes in susceptibility to atopic eczema and to the eczema-associated asthma complex phenotype in children. Furthermore, we examined the interactive effects between these genetic variants and early-life exposure to ETS on the development of the combined asthma–eczema phenotype in an independent population of Polish children.

## 2. Results

A summary of the characteristics of patients with atopic eczema and control subjects, including genotyping results for the *TNS1* rs918949 and *NRXN1* rs10194978 SNPs, as well as information on ETS exposure status, is presented in Table 1. No significant differences were observed between cases and controls with respect to age or sex.

### 2.1. The Association of TNS1 rs918949 and NRXN1 rs10194978 with Eczema

In the overall study population, no significant associations were found between the *TNS1* rs918949 and *NRXN1* rs10194978 polymorphisms and the prevalence of atopic eczema. The eczema and control groups did not differ significantly in genotype frequencies for either *TNS1* rs918949 (*p* = 0.441; χ^2^ = 0.592) or *NRXN1* rs10194978 (*p* = 0.730; χ^2^ = 0.119). In genotype and allele models adjusted for potential confounders, the risk alleles of both SNPs did not significantly increase the risk of atopic eczema (Table 2 and Table 3).

### 2.2. The Association of TNS1 rs918949 and NRXN1 rs10194978 with the Asthma–Eczema Complex Phenotype

Associations between *TNS1* rs918949 and tested phenotypes are summarized in Table 2. Analysis of the *TNS1* rs918949 variant demonstrated that the risk allele [T] was associated with an increased predisposition to the asthma–eczema comorbidity compared with controls without asthma or eczema (*p* = 0.023; χ^2^ = 5.132). This association remained significant when compared with an expanded control group comprising all participants who did not belong to the combined phenotype group (*p* = 0.016; χ^2^ = 5.737). Individuals carrying at least one [T] allele exhibited more than a twofold higher risk of having eczema-associated asthma (OR = 2.00, 95% CI 1.08–3.71; *p* = 0.031) in the allelic analysis. In genotype models adjusted for relevant confounders, individuals homozygous for the effect allele demonstrated a significantly increased risk of the combined asthma–eczema phenotype compared with wild-type homozygotes under the codominant model. This association was also evident—and even stronger—when these individuals were compared with controls without asthma or eczema in the recessive model (OR = 6.75, 95% CI 2.37–19.2; *p* < 0.001). Next, to verify the specificity of the observed association with comorbidity, the analysis was restricted to children with atopic eczema. First, we examined the association between *TNS1* rs918949 and eczema-associated asthma by comparing participants with eczema plus asthma to those with eczema only. A significant association of the *TNS1* rs918949 risk allele with eczema-associated asthma was confirmed at both the genotype and allele levels. In the additive model, the presence of the T allele was associated with an approximately twofold increase in risk (OR = 2.08, 95% CI 1.11–3.88, *p* = 0.028), while in the recessive model, the risk was further increased. The strongest effect was observed in the codominant model, where carriers of the TT genotype showed an increased risk of the asthma–eczema phenotype compared with wild-type homozygotes. Then, the association between *TNS1* rs918949 and eczema was evaluated by comparing participants with eczema only to controls without asthma or eczema. Notably, in the absence of asthma, no significant association with eczema was observed in any of the tested genetic models (Table 2).

No significant associations were observed between the *NRXN1* rs10194978 variant and the asthma–eczema comorbidity in any of the analyses performed. Specifically, genotype and allele frequencies did not differ significantly across the tested phenotypic groups, nor between the subgroups with eczema plus asthma (*p* = 0.548; χ^2^ = 0.360) and eczema only (*p* = 0.991; χ^2^ = 0.018), when compared with the respective control groups. This lack of differences in allelic and genotypic distributions persisted when the control group was expanded to include all participants not belonging to the combined phenotypic group (*p* = 0.558; χ^2^ = 0.342). In the allelic model, carriers of the G allele did not show a significantly increased risk of eczema-associated asthma compared with controls. Similarly, genotype-based analyses under dominant, recessive, and codominant models revealed no significant associations between *NRXN1* rs10194978 and the combined asthma–eczema phenotype after adjustment for relevant confounders. No significant associations were detected in further analysis restricted to children with atopic eczema or in the absence of asthma. (Table 3).

### 2.3. Interaction Analysis of TNS1 rs918949 and NRXN1 rs10194978 with ETS Exposure in Relation to Asthma–Eczema Complex Phenotype

In our study population, exposure to ETS during the first two years of life was significantly more frequent among children with the asthma–eczema comorbidity compared to the control group (*p* = 0.006, χ^2^ = 8.434). In contrast, the frequency of parent-reported early-life ETS exposure did not differ significantly between children with eczema and healthy controls (*p* = 0.272, χ^2^ = 1.347). In logistic regression analysis, early-life ETS exposure was significantly associated with the combined eczema–asthma phenotype (OR = 3.59; 95% CI 1.48–8.74), whereas no such association was observed for eczema alone (OR = 1.46; 95% CI 0.89–2.49).

Next, we explored whether the early-life ETS exposure interacts with *TNS1* and *NRXN1* variants to modulate the risk of developing the combined asthma–eczema phenotype. Table 4 presents additional analyses for the risk of eczema-associated asthma after stratification by ETS exposure status. Among children exposed to ETS, carriers of the *TNS1* rs918949 T allele showed a markedly increased risk of the asthma–eczema comorbidity compared with carriers of the C allele (RR = 8.50, 95% CI 3.02–23.95; *p* < 0.001). In contrast, no association was observed for *TNS1* in the absence of ETS exposure (RR = 0.71, 95% CI 0.29–1.73; *p* = 0.509). Similarly, for *NRXN1* rs10194978, the A allele was associated with an increased risk of eczema-associated asthma among ETS-exposed children (RR = 4.23, 95% CI 1.62–11.52; *p* = 0.005), whereas no association was detected in non-exposed children (RR = 1.02, 95% CI 0.41–2.59; *p* = 1.000). Therefore, ETS exposure was included as a secondary predictor in a logistic regression model to evaluate its potential confounding effect. After adjustment for ETS exposure, the *TNS1* rs918949 risk allele remained significantly associated with the asthma–eczema phenotype (OR = 2.20, 95% CI 1.15–4.21; *p* = 0.016), indicating little evidence of confounding by ETS exposure. For the *NRXN1* rs10194978 variant, the association of the risk allele [A] also remained significant after adjustment (OR = 2.14, 95% CI 1.10–4.28; *p* = 0.031). However, early-life ETS exposure appeared to confound this relationship, as the adjusted OR was higher than the crude OR for the *NRXN1*–asthma–eczema association. Furthermore, early-life ETS exposure emerged as an important independent predictor of the combined asthma–eczema phenotype, even after controlling for both *TNS1* rs918949 and *NRXN1* rs10194978 risk alleles with adjusted OR = 3.81, 95% CI 2.00–7.25; *p* < 0.001 and OR = 4.45, 95% CI 2.27–8.72; *p* < 0.001, respectively (Table 4).

To investigate potential gene–environment interactions, we examined the combined effects of early-life ETS exposure and *TNS1* and *NRXN1* variants on the risk of developing the asthma–eczema phenotype. Significant interactions were observed for both *TNS1* rs918949 and *NRXN1* rs10194978, indicating that early-life ETS exposure enhanced the genetic risk associated with the respective effect alleles. Specifically, carriers of at least one risk allele of either *TNS1* rs918949 or *NRXN1* rs10194978 who were exposed to ETS had the highest risk of developing the combined asthma–eczema phenotype compared with nonexposed children carrying the wild-type genotype (Table 5).

The measures of interaction (RERI, AP, and synergy index) indicated a significant positive interaction on the additive scale, suggesting that the combined effect of both risk factors was significantly greater than the sum of their individual effects. Furthermore, the risk of the eczema-associated asthma phenotype among exposed children carrying the risk alleles fitted a multiplicative model, with ratios of relative risks of 4.70 for *TNS1* and 2.16 for *NRXN1*, respectively (Table 5).

A logistic regression model including an interaction term between genotype and ETS exposure in relation to the asthma–eczema comorbidity confirmed a significant gene–environment interaction for both *TNS1* rs918949 (*p* for interaction < 0.001) and *NRXN1* rs10194978 (*p* for interaction = 0.037), consistent with their synergistic effect on the risk of developing the combined asthma–eczema phenotype.

Post hoc power analysis indicated moderate power to detect the main genetic effect of *TNS1* rs918949 (achieved power ≈ 0.59), whereas power to detect the main effect of *NRXN1* rs10194978 was low (achieved power ≈ 0.20). In contrast, power to detect genotype × ETS interaction effects was high for *TNS1* (achieved power > 0.90) and moderate for *NRXN1* (achieved power ≈ 0.55), likely driven by the relatively large observed effect sizes for the interaction terms (Table S1).

## 3. Discussion

In this study, we evaluated whether early-life exposure to ETS interacts with genetic variants at two candidate loci, *TNS1* and *NRXN1*, to impact the development of the combined asthma–eczema phenotype in children. These loci were previously identified in the only existing genome-wide interaction study of this phenotype, where both demonstrated significant interaction signals with ETS [36]. In addition, we extended these analyses by evaluating the associations of *TNS1* and *NRXN1* variants with susceptibility to atopic eczema and to the eczema-associated asthma phenotype in children, irrespective of ETS exposure. We found that *TNS1* rs918949 was associated with an increased risk of the asthma–eczema phenotype, and gene–environment interaction analyses indicated that both *TNS1* and *NRXN1* variants conferred the greatest risk among children exposed to ETS in early life.

To the best of our knowledge, there are no published data linking the *TNS1* rs918949 variant to asthma–eczema comorbidity, a phenotype that has been rarely investigated and is generally overlooked in most genetic studies. To date, only three GWAS meta-analyses focusing on this combined phenotype have been reported. The first, examining eczema followed by asthma within the framework of the atopic march, identified five loci previously associated with at least one allergic disease (*FLG*, *IL4/KIF3A*, *AP5B1/OVOL1*, *C11orf30/LRRC32*, and *IKZF3*) and two novel loci specific to the eczema–asthma phenotype (*EFHC1* and *TMTC2*) [16]. A subsequent GWAS meta-analysis designed to detect genes uniquely contributing to this comorbidity phenotype revealed six new loci not previously linked to allergic disease, including two SNPs in the *OAC2* gene that reached genome-wide significance [17]. Most recently, a GWAS conducted in a Korean pediatric population identified *CACNA2D3* (3p14.3) as a novel locus, with the lead variant showing an association with reduced risk of asthma and atopic dermatitis multimorbidity [37]. Together, these findings support the view that the asthma–eczema comorbidity represents a distinct biological phenotype, defined by genetic determinants that only partially overlap with those involved in isolated asthma or eczema. Our results are broadly consistent with this concept, as we demonstrate that the *TNS1* rs918949 variant is specifically associated with the combined asthma–eczema phenotype but not with eczema alone. The increased asthma risk associated with the rs918949 [T] allele observed among children with eczema argues against the interpretation that the association with the asthma–eczema comorbidity merely reflects susceptibility to eczema alone. This is further supported by the absence of any association between *TNS1* rs918949 and eczema in the absence of asthma. Together, these findings suggest that *TNS1* may act as a susceptibility locus predominantly in the context of the combined asthma–eczema phenotype. However, due to the study design, all asthmatic participants in our cohort also presented with eczema, which precluded the evaluation of genetic associations with asthma in the absence of eczema. Consequently, we cannot determine whether the observed association is specific to the asthma–eczema comorbidity or only reflects a more general effect on asthma risk. Therefore, extrapolation of these findings to asthma phenotypes without concomitant eczema should be made with caution. Taken together, these results are consistent with the hypothesis that asthma–eczema comorbidity may reflect a distinct immunobiological phenotype rather than a simple coexistence of two independent conditions.

Although the precise biological mechanisms linking *TNS1* to allergic multimorbidity have not yet been elucidated, several lines of evidence suggest that this gene may play a role in pathways relevant to epithelial barrier integrity and tissue remodeling—key processes in both eczema and asthma pathophysiology [38,39,40]. *TNS1* encodes tensin-1, a focal adhesion protein that links the extracellular matrix components to the actin cytoskeleton, participates in various signaling pathways, and thereby influences cell adhesion, migration, and proliferation, as well as epithelial integrity and cytoskeletal remodeling [41]. In the human lung, *TNS1* has been detected in airway smooth muscle, the lamina propria, and the airway epithelium, and its expression is markedly upregulated in response to TGF-β (Transforming growth factor beta), a key cytokine involved in inflammation and fibrotic airway remodeling of asthmatic patients [42,43]. Functional studies further demonstrated that *TNS1* regulates α-smooth muscle actin expression and contractile responses in human airway smooth muscle cells, directly implicating this protein in asthma-related airway pathology [43]. In line with these functional observations, previous GWASs have identified significant associations between *TNS1* genetic variants and spirometric measures, including post-bronchodilator FEV_1_ and the FEV_1_/FVC ratio (Forced expiratory volume in one second/Forced vital capacity), suggesting a potential role for *TNS1* in pulmonary function and respiratory pathophysiology [44,45]. In skin-related contexts, *TNS1* is expressed in human dermal fibroblasts, where it is thought to contribute to the stability, tension, and structural organization of the extracellular matrix mediated by fibroblasts [46,47,48]. Experimental studies showed that reduced *TNS1* expression impairs cell–matrix interactions at both focal and fibrillar adhesions, supporting a functional role for TNS1 in maintaining extracellular matrix architecture and overall dermal structural integrity [47,49]. Against this background, the observed association of the *TNS1* rs918949 variant with the asthma–eczema comorbidity appears biologically plausible, suggesting that genetic variation within *TNS1* may modulate barrier-related pathways, particularly relevant in multimorbid allergic disease.

A key finding of this study is the significant interaction observed between the *TNS1* rs918949 and *NRXN1* rs10194978 variants and early-life ETS exposure in relation to the asthma–eczema comorbidity phenotype. Children exposed to ETS during the first two years of life who carried risk alleles at either locus exhibited the highest susceptibility to the combined phenotype. The observed interactions suggest a synergistic model in which genetic predisposition and ETS exposure jointly increase disease risk beyond what would be expected from their individual contributions. These findings are consistent with the only GWIS meta-analysis of asthma and eczema published to date, which considers ETS exposure in four independent populations. This study resulted in the detection of four new SNPs, among which *TNS1* and *NRXN1* showed significant evidence for their interaction with ETS exposure in relation to the comorbidity of asthma and eczema [36]. Our study provides the first independent replication of these findings in a case-control setting, further supporting the hypothesis that *TNS1* and *NRXN1* contribute to the susceptibility underlying the asthma–eczema phenotype and that early-life environmental exposures modulate their effects. Additional support for this interpretation comes from GWAS and association studies demonstrating interactions between active or passive smoking and *TNS1* variants in relation to pulmonary function in adults, as well as transient wheezing and lung function in early childhood [50,51,52]. Likewise, genetic variation in *NRXN1* has previously been associated with lung-related phenotypes and spirometric measures, particularly in current and former smokers, suggesting a potential role of this locus in pulmonary function and environmentally responsive respiratory pathways [53]. In the present study, *NRXN1* did not show a significant main effect in unstratified analyses, and the observed association emerged primarily in the context of ETS exposure. This pattern is consistent with, but does not definitively establish, a context-dependent genetic effect in which genetic susceptibility may become apparent only under specific environmental conditions [54]. However, given the absence of a main genetic effect, the moderate statistical power for the *NRXN1* × ETS interaction, and the relatively wide confidence intervals, it cannot be ruled out that factors such as exposure misclassification or limited sample size within exposure strata contributed to these findings. Although neurexins encoded by the *NRXN1* gene are traditionally recognized for their roles in neurotransmission and synaptic function, emerging evidence indicates that they are also expressed in epithelial cells and participate in cell adhesion and developmental signaling pathways, which are known to be susceptible to environmental influences and may therefore underlie the ETS-dependent effects [55,56,57,58,59]. Nevertheless, these results should be interpreted as preliminary, and the biological relevance of *NRXN1* and ETS exposure on the risk of eczema-asthma comorbidity remains speculative at this stage. Accordingly, our findings should be interpreted as hypothesis-generating rather than confirmatory, warranting replication in larger cohorts with more detailed exposure assessment, as well as functional studies to elucidate the underlying molecular mechanisms. Despite these considerations, these observations highlight the importance of considering environmental modulators when evaluating genetic risk, particularly in complex atopic conditions.

More broadly, our findings are consistent with the established concept of gene–environment interaction, in which genetic susceptibility and environmental exposures jointly influence disease risk by modifying each other’s effects [54]. In this context, it is conceivable that early-life ETS enhances the effect size of the risk alleles at *TNS1* and *NRXN1*, while conversely, these genetic variants may shape the extent to which ETS exposure increases the risk of eczema-associated asthma. From a pathophysiological perspective, it is plausible that ETS interacts with underlying DNA sequence variation through mechanisms such as modulation of enhancer or promoter activity, chromatin remodeling, and activation of specific transcription factors, ultimately leading to differential gene expression [60,61,62,63,64,65]. For completeness, beyond DNA sequence-dependent interactions, components of ETS are also known to induce a range of epigenetic modifications—including changes in DNA methylation, post-translational histone modifications, and regulation of non-coding RNA expression—which can influence gene expression and phenotype without altering the DNA sequence [66,67,68]. Within the broader framework of gene–environment interaction, it can be assumed that genetic variants in *TNS1* and *NRXN1*, involved in epithelial integrity and cell adhesion, may heighten susceptibility to the detrimental effects of early-life ETS exposure. It is well established that chemicals present in tobacco smoke can trigger the immune system, modulate innate and adaptive immune responses, and promote inflammatory processes, including allergic inflammation and sensitization [18,19,20,68,69,70,71]. The tobacco-related toxins disrupt airway epithelial differentiation and integrity, impair mucociliary function, enhance mucous production, and increase oxidative stress, leading to activation of the inflammatory response, further lung tissue damage, and airway remodeling [72,73,74,75,76,77,78,79,80,81]. Chemical constituents of tobacco smoke also exert multiple detrimental effects on the skin barrier and extracellular matrix [82,83]. Experimental studies demonstrate that ETS exposure increases transepidermal water loss, promotes degeneration of dermal connective tissue, and induces the upregulation of matrix metalloproteinases—particularly MMP-1 and MMP-3 (Matrix metalloproteinases)—which accelerate the degradation of collagen and elastic fibers [83,84]. Additionally, compounds present in tobacco smoke have been shown to facilitate epicutaneous sensitization by promoting Langerhans cell migration, inducing the release of epithelial alarmins such as TSLP (Thymic stromal lymphopoietin) and IL-33, and enhancing Th2- and Th17-skewed immune responses, suggesting a plausible mechanism through which ETS exposure may contribute to the development of atopic eczema [85,86].

Taken together, these mechanistic insights align with epidemiological observations demonstrating that early-life ETS exposure is a robust risk factor for asthma-related outcomes and may also contribute to the development of atopic eczema, particularly in genetically susceptible children. Numerous cohort and observational studies consistently show that both prenatal and postnatal ETS exposure are associated with impaired lung development, increased wheezing, reduced lung function, and a higher risk of developing asthma [21,22,87,88,89,90,91,92,93]. In contrast, the evidence linking ETS exposure to atopic eczema is more heterogeneous: although several studies report elevated risk with active or passive smoking, the overall findings remain less consistent [23,24,25,34]. In our study, early-life ETS exposure emerged as an independent predictor of the combined asthma–eczema phenotype, even after adjustment for genetic variation. By contrast, ETS exposure did not significantly increase the risk of eczema alone, suggesting that environmental exposures may preferentially influence developmental pathways leading to the expression of specific phenotypes. It can be assumed that ETS may act as a “second hit” that facilitates the transition from skin inflammation to respiratory manifestations, consistent with models of sequential allergic disease progression.

The early-life timing of ETS exposure is particularly noteworthy. The first two years of life represent a critical window for gene–environment interactions, as the lungs, skin, epithelial barrier, immune, and neuroendocrine systems still undergo rapid maturation, rendering infants especially vulnerable to the adverse effects of ETS that may exert long-lasting impact [94,95,96]. These considerations highlight the need for further research focusing on early-life gene–environment interactions in pediatric populations, an area that remains insufficiently explored despite persistently high levels of tobacco smoke exposure in children. Another implication of our work is that studying precise phenotypes and comorbidities is a critical feature in genetic research, especially when investigating gene–environment interactions, which often manifest in phenotype-specific ways.

The strengths of this study include its prospective design, which enabled repeated monitoring of environmental exposures and clinical outcomes within the same cohort; detailed phenotypic characterization; and the use of well-matched healthy controls recruited from the general population. The independent replication of interaction signals previously reported in a GWIS further adds credibility to our findings and demonstrates the utility of targeted replication in hypothesis-driven gene–environment research. However, several limitations should be acknowledged. The relatively small sample size reduces statistical power and increases the likelihood of both false-negative findings and spurious false-positive associations, particularly for interaction analyses. Statistical power in gene–environment analyses is typically lower than in main-effect genetic analyses, particularly when environmental exposures are dichotomized and unevenly distributed across subgroups, as was the case for early-life ETS in our cohort. Consistent with this, post hoc power analysis indicated moderate-to-low power for detecting main genetic effects, particularly for *NRXN1*, which should be considered when interpreting null findings. ETS exposure was assessed by parental self-report and may therefore affect the precision of interaction effect estimates by introducing measurement error, including recall and social desirability bias. Moreover, detailed information on exposure intensity, duration, and prenatal timing was unavailable. While this approach is commonly used in pediatric epidemiological studies and enables the inclusion of environmental factors when more detailed exposure data are lacking, it may introduce non-differential exposure misclassification. Such misclassification is expected to bias effect estimates toward the null and further reduce statistical power, particularly for detecting gene–environment interactions. Consequently, the observed genotype × ETS interactions may represent conservative estimates of the true effects. At the same time, the use of a dichotomous exposure variable precludes evaluation of dose–response relationships and limits inference regarding critical exposure windows. In addition, other environmental factors that may interact with genetic susceptibility were not captured. Despite these limitations, post hoc analyses suggested comparatively higher power for detecting genotype × ETS interaction effects, likely driven by the relatively large interaction effect sizes observed in the present sample. Nevertheless, given the limited number of children with the asthma–eczema phenotype, the magnitude of these effects should be interpreted with caution. However, these considerations do not diminish the biological plausibility of the observed interactions. Future studies in larger, independent cohorts incorporating quantitative, time-resolved, and prenatal exposure measures, ideally supported by objective biomarkers, as well as functional studies aimed at elucidating the underlying biological mechanisms, are warranted to confirm and refine these findings.

In conclusion, this study provides evidence that genetic variation in *TNS1* and *NRXN1* may interact synergistically with early-life ETS exposure to increase susceptibility to the combined asthma–eczema phenotype in children. The context-dependent nature of these genetic effects highlights the importance of including gene–environment interactions in studies of complex allergic diseases. We identified *TNS1* rs918949 as a locus specifically linked to the comorbid phenotype but not with eczema alone, supporting the idea that asthma–eczema multimorbidity is a distinct biological phenotype rather than the coexistence of two separate conditions. Early-life ETS exposure also emerged as an independent predictor of the combined phenotype, underscoring the importance of considering environmental factors when assessing genetic susceptibility in complex atopic conditions. From a prevention standpoint, our findings suggest that early childhood is a critical developmental period during which ETS avoidance may be particularly crucial, especially for children with a genetic predisposition. Future replication studies in larger groups, detailed exposure assessments, and functional analyses of the involved pathways will be necessary to confirm these results and better understand how early-life exposures interact with genetic factors to influence asthma–eczema comorbidity.

## 4. Materials and Methods

### 4.1. Study Population

A total of 188 unrelated children (107 males), all younger than 2 years at the time of recruitment, were enrolled in the study. This group included 103 patients with atopic eczema (mean age: 13.2 ± 6.7 months) and 85 healthy control subjects (mean age: 15.3 ± 5.6 months). All participants were prospectively followed at yearly intervals until reaching 6 years of age. The entire study population was of Caucasian ethnicity. The study subjects were recruited from patients attending the Outpatient Clinic for Children at Wroclaw Medical University Hospital and from the general population. Recruitment of the control group was community-based and involved the dissemination of flyers in local nurseries, pediatric and family medicine practices, and during health fairs. Parents who expressed interest were invited to contact the research coordinator by phone. Eligibility of all participants, both cases and controls, was assessed using a structured questionnaire designed to collect comprehensive information on general health status, symptoms of atopic eczema or other allergic diseases, sociodemographic data, exposure to environmental factors including tobacco smoke, and family history of allergic conditions. The subjects with atopic eczema were examined and diagnosed in accordance with the diagnostic criteria proposed by Hanifin and Rajka [97]. The mean (±SD) age at disease onset was 4.6 ± 3.5 months. Asthma was defined as physician-diagnosed asthma ever by the age of 6 years. Asthma at 6 years of age was defined by the presence of a previous asthma diagnosis made by a doctor during follow-up visits and one or more wheezing episodes during the 12 months preceding the analysis. Eczema-associated asthma at 6 years of age was defined by the presence of a previous physician’s diagnosis of eczema according to the criteria established by Hanifin and Rajka, visible eczema at the time of follow-up together with the presence of a previous asthma diagnosis made by a doctor during the follow-up visits, and 1 or more wheezing episodes during the 12 months before the analysis. The control group, matched to the case group for age and sex, consisted of healthy children who met the following inclusion criteria: absence of any clinical symptoms of atopic eczema and asthma, and no reported family history of allergic diseases.

Exposure to environmental tobacco smoke (ETS) during early childhood was defined as a positive response by the child’s mother or father to the question, “Did you or your child’s other parent smoke when your child was younger than 2 years of age?”

Serum samples obtained from all participants were analyzed for total and allergen-specific IgE concentrations. The panel of specific IgE measurements included the 10 most common inhalant and 10 most common food allergens. Total serum IgE concentrations were determined using a commercial chemiluminescent immunoassay (IMMULITE 2000 Total IgE; Diagnostic Products Corporation, Los Angeles, CA, USA). Allergen-specific IgE levels were assessed using a standardized enzyme immunoassay (Polycheck; BIOCHECK, Munster, Germany). Allergic sensitization was defined as the presence of specific IgE to at least one of the tested allergens at a concentration ≥ 0.7 kU/L (class II or higher).

### 4.2. Genotyping

Genotyping for the single-nucleotide polymorphisms (SNPs) rs918949 and rs10194978, located in the *TNS1* (2q35) and *NRXN1* (2p16) genes, respectively, was performed in 188 subjects. Genomic DNA was extracted from EDTA-anticoagulated whole blood samples using the QIAamp DNA Blood Mini Kit (QIAGEN GmbH, Hilden, Germany) according to the manufacturer’s instructions. All genotyping assays were conducted using the LightSNiP assay (TibMolbiol, Berlin, Germany). Polymerase chain reaction (PCR) amplification was carried out in a final volume of 10 µL containing 1 µL of genomic DNA (15–60 ng/µL), 0.5 µL of reagent mix containing specific primers and SimpleProbe^®^ probes at optimized concentrations, 0.8 µL of MgCl_2_, and 1 µL of LightCycler^®^ FastStart DNA Master HybProbe (Roche Applied Science, Mannheim, Germany). Reactions were performed on a LightCycler 1.5 platform (Roche Applied Science, Mannheim, Germany). For quality assurance, positive controls representing each genotype, as well as negative (no-template) controls, were included in every run.

### 4.3. Statistical Analysis

The Hardy–Weinberg equilibrium was assessed among control subjects using the χ^2^ goodness-of-fit test to compare observed and expected genotype frequencies. Differences in genotype distributions and demographic characteristics between case and control groups were analyzed using the χ^2^ test or Fisher’s exact test, as appropriate. Associations between genotypes or alleles and disease status were evaluated by calculating odds ratios (ORs) with corresponding 95% confidence intervals (CIs) and *p*-values, using logistic regression analysis. Both crude and adjusted ORs were estimated, with adjustments made for age, sex, and family history of atopy. Statistical significance was defined as a *p*-value < 0.05. Associations between SNPs and disease phenotypes were evaluated under additive, dominant, recessive, and codominant models of inheritance using logistic regression analyses adjusted for potential confounders. The χ^2^ test or Fisher’s exact test was applied to assess the combined effect of genotype pairs. Gene–environment interactions between the tested SNPs and early-life exposure to tobacco smoke in relation to the combined asthma–eczema were assessed using logistic regression models including interaction terms (SPSS). To determine whether an interaction between two risk factors (A and B) was present, the relative excess risk due to interaction (RERI), the attributable proportion due to interaction (AP), the synergy index (S), and the ratio of relative risks (RRs) were calculated, as recommended by Knol et al. [98,99]. Interaction was defined as a deviation from either the additive or multiplicative model. On the additive scale, RERI and AP values greater than zero indicated a superadditive (positive) interaction, whereas values less than zero indicated a subadditive (negative) interaction. Similarly, an S value greater than 1 indicated a superadditive effect, while an S value less than 1 indicated a subadditive effect. On the multiplicative scale, a ratio of ORs greater than 1 suggested a positive interaction, whereas a ratio less than 1 indicated a negative interaction. Post hoc power calculations were performed for both main genetic effects and gene–environment interaction effects using Wald-based normal approximations derived from the logistic regression models. Odds ratios were transformed to the log-odds scale (β = ln [OR]), and standard errors were obtained from the reported 95% confidence intervals or directly from the regression output. Achieved power for two-sided tests at α = 0.05 was approximated as Φ (|β|/SE − 1.96), where Φ denotes the standard normal cumulative distribution function. All statistical analyses were performed using STATISTICA software, version 9.0 (StatSoft Inc., Tulsa, OK, USA), and SPSS Statistics software, version 11.1 (SPSS Inc., Chicago, IL, USA).

The study received approval from the Ethics Committee of Wroclaw Medical University, Wroclaw, Poland (protocol codes: 392/11, 631/21, and 264/24), and informed written consent, including consent for genetic studies, was obtained from all subjects before they participated in the testing.

## Figures and Tables

**Table 1:** Characteristics of the study group.

Variable	Atopic Eczema	Control
Age at the time of recruitment, month (mean ± SD)	13.6 ± 6.7	15.9 ± 5.6
Gender (male/female)	63/40	44/41
Allergic sensitization (%)	55 (53.4%)	11 (12.9%)
Asthma (%)	28 (27.2%)	0
Atopic hereditary (%)	57 (55.3%)	0
Serum Total IgE, IU/mL, geometric mean, 95% CI	24.6 (27.8 ÷ 53.4)	17.7 (14.3 ÷ 22.2)
*TNS1* rs918949		
CC	33 (32.0%)	24 (28.2%)
CT	42 (40.8%)	48 (56.5%)
TT	28 (27.2%)	13 (15.3%)
*NRXN1* rs10194978		
GG	21 (20.4%)	20 (23.5%)
GA	45 (43.7%)	35 (41.2%)
AA	37 (35.9%)	30 (35.3%)
Tobacco smoke exposure:		
YES	36 (34.9%)	23 (27.1%)
NO	67 (65.1%)	62 (72.9%)

**Table 2:** Associations between *TNS1* rs918949 genotype and allergic diseases. The control * group comprises all participants who did not belong to the combined phenotype group.

*TNS1* rs918949 Genotype Status	Genotype/ Risk Allele Frequency Total *n* (%)	Phenotype				
		Eczema vs. Control	Asthma Plus Eczema vs. Control	Asthma Plus Eczema vs. Control *	Asthma Plus Eczema vs. Eczema Only	Eczema Without Asthma vs. Control
Total *n* (%)		103/188 (54.8%)	28/113 (24.8%)	28/188 (14.9%)	28/103 (27.2%)	75/160 (46.9%)
CC CT TT	57/188 (30.3%) 90/188 (47.9%) 41/188 (21.8%)	1.0 Reference *p* = 0.236 0.64 (0.32 ÷ 1.24) *p* = 0.398 1.56 (0.68 ÷ 3.64)	1.0 Reference *p* = 0.776 0.83 (0.27 ÷ 2.56) *p* = 0.043 3.69 (1.12 ÷ 12.1)	1.0 Reference *p* = 1.000 1.06 (0.36 ÷ 3.10) *p* = 0.032 3.52 (1.19 ÷ 10.36)	1.0 Reference *p* = 0.586 1.41 (0.45 ÷ 4.37) *p* = 0.050 3.75 (1.08 ÷ 10.7)	1.0 Reference *p* = 0.155 0.59 (0.29 ÷ 1.20) *p* = 1.000 1.09 (0.44 ÷ 2.73)
CC vs. CT + TT	131/188 (69.7%)	*p* = 0.634 0.83 (0.44 ÷ 1.56)	*p* = 0.623 1.44 (0.52 ÷ 3.99)	*p* = 0.373 1.72 (0.66 ÷ 4.49)	*p* = 0.235 2.06 (0.74 ÷ 5.71)	*p* = 0.312 0.69 (0.36 ÷ 1.36)
CC + CT vs. TT	41/188 (21.8%)	*p* = 0.072 2.07 (0.99 ÷ 4.30)	*p* < 0.001 6.75 (2.37 ÷ 19.2)	*p* = 0.006 3.39 (1.45 ÷ 7.92)	*p* = 0.045 2.76 (1.10 ÷ 7.01)	*p* = 0.411 1.50 (0.67 ÷ 3.72)
C vs. T	172/376 (45.7%)	*p* = 0.467 1.18 (0.78 ÷ 1.77)	*p* = 0.031 2.00 (1.08 ÷ 3.71)	*p* = 0.020 2.04 (1.14 ÷ 3.64)	*p* = 0.028 2.08 (1.11 ÷ 3.88)	*p* = 0.910 0.96 (0.62 ÷ 1.50)

**Table 3:** Associations between *NRXN1* rs10194978 genotype and allergic diseases. The control * group comprises all participants who did not belong to the combined phenotype group.

NRXN1 rs10194978 Genotype Status	Genotype/ Risk Allele Frequency Total *n* (%)	Phenotype				
		Eczema vs. Control	Asthma Plus Eczema vs. Control	Asthma Plus Eczema vs. Control *	Asthma Plus Eczema vs. Eczema Only	Eczema Without Asthma vs. Control
Total *n* (%)		103/188 (54.8%)	28/113 (24.8%)	28/188 (14.9%)	28/103 (27.2%)	75/160 (46.9%)
GG GA AA	41/188(21.8%) 80/188(42.6%) 67/188(35.6%)	1.0 Reference *p* = 0.700 1.22 (0.57 ÷ 2.61) *p* = 0.696 1.17 (0.54 ÷ 2.56)	1.0 Reference *p* = 0.388 2.00 (0.58 ÷ 6.91) *p* = 0.541 1.67 (0.46 ÷ 6.06)	1.0 Reference *p* = 0.295 1.96 (0.60 ÷ 6.39) *p* = 0.561 1.62 (0.47 ÷ 5.59)	1.0 Reference *p* = 0.383 1.92 (0.54 ÷ 6.76) *p* = 0.544 1.57 (0.43 ÷ 5.83)	1.0 Reference *p* = 1.000 1.04 (0.46 ÷ 2.34) *p* = 1.000 1.06 (0.46 ÷ 2.43)
GG vs. GA + AA	147/188 (78.2%)	*p* = 0.723 1.20 (0.60 ÷ 2.40)	*p* = 0.426 1.85 (0.57 ÷ 5.95)	*p* = 0.456 1.81 (0.59 ÷ 5.43)	*p* = 0.420 1.76 (0.54 ÷ 5.77)	*p* = 1.000 1.05 (0.50 ÷ 2.19)
GG + GA vs. AA	67/188 (35.6%)	*p* = 1.000 1.03 (0.56 ÷ 1.87)	*p* = 1.000 1.02 (0.42 ÷ 2.48)	*p* = 1.000 1.01 (0.43 ÷ 2.32)	*p* = 1.000 0.99 (0.36 ÷ 2.44)	*p* = 1.000 1.03 (0.54 ÷ 1.97)
G vs. A	211/376 (56.1%)	*p* = 0.834 1.05 (0.69 ÷ 1.58)	*p* = 0.280 1.42 (0.76 ÷ 2.65)	*p* = 0.306 1.40 (0.78 ÷ 2.52)	*p* = 0.344 1.38 (0.73 ÷ 2.59)	*p* = 0.910 1.03 (0.66 ÷ 1.61)

**Table 4:** Eczema-associated asthma risk after stratification for early-life ETS exposure.

Genotype	ETS Exposure (Yes) *p*-Value RR (95% CI)	ETS Exposure (No) *p*-Value RR (95% CI)
*TNS1* rs918949		
*TNS1* rs918949 allele C	1.00 (Reference) -	1.00 (Reference) -
*TNS1* rs918949 allele T	*p* < 0.001 8.50 (3.02 ÷ 23.95)	*p* = 0.509 0.71 (0.29 ÷ 1.73)
*NRXN1* rs10194978		
*NRXN1* rs10194978 allele G	1.00 (Reference) -	1.00 (Reference) -
*NRXN1* rs10194978 allele A	*p* = 0.005 4.23 (1.62 ÷ 11.52)	*p* = 1.000 1.02 (0.41 ÷ 2.59)

**Table 5:** Interaction between *TNS1* rs918949 and *NRXN1* rs10194978 and early-life ETS exposure in eczema-associated asthma. RERI (the relative excess risk due to interaction), AP (the attributable proportion due to interaction), S (the synergy index).

Genotype and Exposure Combinations	Eczema-Associated Asthma *n* (%)	Control *n* (%)	*p*-Value	RR (95% CI)	
*TNS1* rs918949 allele C ETS exposure (No)	14 (25.0%)	62 (36.5%)	-----	1.00 (Reference)	RERI = 2.84; AP = 0.78; S = 52.4 ratio of RRs = 4.70
*TNS1* rs918949 allele T ETS exposure (No)	10 (17.8%)	62 (36.5%)	*p* = 0.509	0.75 (0.33 ÷ 1.69)	
*TNS1* rs918949 allele C ETS exposure (Yes)	8 (14.3%)	34 (20.0%)	*p* = 1.000	1.03 (0.42 ÷ 2.40)	
*TNS1* rs918949 allele T ETS exposure (Yes)	24 (42.9%)	12 (7.0%)	*p* < 0.001	3.62 (2.10 ÷ 6.07)	
*NRXN1* rs10194978 allele G ETS exposure (No)	8 (14.3%)	42 (24.7%)	-----	1.00 (Reference)	RERI = 2.07; AP = 0.55; S = 2.38 ratio of RRs = 2.16
*NRXN1* rs10194978 allele A ETS exposure (No)	16 (28.6%)	82 (48.2%)	*p* = 1.000	1.05 (0.46 ÷ 2.56)	
*NRXN1* rs10194978 allele G ETS exposure (Yes)	12 (21.4%)	33 (19.4%)	*p* = 0.220	1.67 (0.69 ÷ 4.16)	
*NRXN1* rs10194978 allele A ETS exposure (Yes)	20 (35.7%)	13 (7.7%)	*p* < 0.001	3.79 (1.85 ÷ 8.14)	

## Data Availability

The original contributions presented in this study are included in the article/Supplementary Materials. Further inquiries can be directed to the corresponding author.

## Supplementary Materials

The following supporting information can be downloaded at: https://www.mdpi.com/article/10.3390/ijms27010346/s1.

## Abbreviations

The following abbreviations are used in this manuscript:

ETS	Environmental tobacco smoke
TNS1	Tensin-1
NRXN1	Neurexin 1
GWAS	Genome-wide association study
GWIS	Genome-wide interaction study
SNP	Single nucleotide polymorphism
OR	Odds ratio
CI	Confidence interval
RERI	Relative excess risk due to interaction
AP	Attributable proportion due to interaction
S	Synergy index
RR	Ratio of relative risk
FLG	Filaggrin—filament aggregating protein
IL-	Interleukins
KIF3A	Kinesin Family Member 3A
AP5B1	Adaptor-Related Protein Complex 5 Subunit Beta 1
OVOL1	Ovo-like transcriptional repressor 1
LRRC32	Leucine-Rich Repeat Containing 32
IKZF3	IKAROS Family Zinc Finger 3
EFHC1	EF-hand domain containing 1
TMTC2	Transmembrane O-Mannosyltransferase Targeting Cadherins 2
OAC2	Aconitase-2
CACNA2D3	Gene encoding a member of the alpha-2/delta subunit family
TGF-β	Transforming growth factor beta
FEV_1_	Forced expiratory volume in one second
FVC	Forced vital capacity
DNA	Deoxyribonucleic acid
MMP	Matrix metalloproteinases
TSLP	Thymic stromal lymphopoietin
