# Supplementary material for: Interactive Effects of Genetic Susceptibility and Early-Life Tobacco Smoke Exposure on the Asthma–Eczema Complex Phenotype in Children: 6-Year Follow-Up Case-Control Study
Source: Int J Mol Sci. 2025 Dec 29;27(1):346. doi: 10.3390/ijms27010346 (PMC12785580; doi:10.3390/ijms27010346)
Supplement: Supplementary file 1 [file ijms-27-00346-s001.zip › ijms-4044299-supplementary.pdf]

Gene	Effect	OR	95% CI	p-value	β = ln(OR)	SE	Achieved power (α = 0.05, two-sided)
**TNS1**	Main effect (allele T vs C)	2.00	1.08–3.71	0.031	0.693	0.315	**0.59**
**TNS1**	Genotype × smoking interaction	11.90	3.05–46.47	0.0004	2.477	0.695	**0.95**
**NRXN1**	Main effect (allele G vs A)	1.42	0.76–2.65	0.280	0.351	0.319	**0.20**
**NRXN1**	Genotype × smoking interaction	4.13	1.09–15.70	0.037	1.418	0.681	**0.55**

Table S1: Power estimated post hoc based on observed effect sizes from logistic regression models.

Power was estimated post hoc using Wald-based normal approximations based on observed odds ratios from logistic regression models. Detailed methodology is provided in the Methods section.
